# Shell-Fish and Enteric Fever

**Published:** 1903-08-15

**Authors:** 


### The Hospital.

A Journal of

The Medical Sciences and Hospital Administration.

Vol. XXXIV.—No. 881. AUGUST 15, 1903.

### Shell-fish and Enteric Fever.

A period of the year during which there is no "r" in the name of the month, and in which, therefore, according to common usage, oysters are not likely to be consumed, is perhaps a favourable time for considering the risks which may possibly be attendant upon eating them when the season for doing so shall have returned. The recent publication of Dr. Timbrell Bulstrode's report to the Local Government Board, on the illnesses which followed the mayoral banquets of last winter at Winchester and at Southampton, illnesses by which, it will be remembered, valuable lives, with that of the Dean of Winchester among them, were sacrificed, affords material from which something of the present state of the trade in oysters and other shell-fish may be gathered; and from which an estimate may be formed of the degree of confidence which may be reposed in the statements of tradesmen with regard to the sources of supply of which they avail themselves. The first alarm with regard to oysters as carriers of enteric fever contagion was given about ten years ago by Sir William Broadbent, in whose practice a series of cases bearing this interpretation had occurred, cases of which one at least terminated fatally; and a speedy accumulation of similar examples led before long to an official examination of oyster beds in general, and to the condemnation of some of them as places which were exposed to sewage contamination and to all the risks which it may from time to time imply. Among the beds then inspected were those of Emsworth, a hamlet of the Warblington Urban District in Hampshire, situate about a mile south-east of Havant. The ponds there received several varieties of oysters, brought from abroad or from the deep sea, and stored until they were required for market, so as to render Emsworth an important distributing centre for oysters of many kinds. The ponds were inspected by Dr. Timbrell Bulstrode in 1895, and he then pointed out the dangerous position which they occupied in relation to the Emsworth sewerage, with the result that certain drains which then discharged directly among the ponds were intercepted and led to the main outfall. The improvement thus effected was a very partial and imperfect one; the present condition of the place being that the sewers are for the most part deficient in fall and leaky, that they are neither properly flushed nor adequately ventilated, and that they discharge mainly by means of one outfall, which is situate in the channel of the estuary, near to the oyster ponds, and which consists of an iron pipe, the end of which is covered with an iron flap. In addition to this outfall there are others discharging into the River Ems at different points of its course. Dr. Bulstrode reported in 1895 that "it would hardly seem necessary, in view of the relations between the sewer outlets and the storage pits, to dwell upon the gross contaminations to which oysters here stored must be liable." The conditions thus referred to have been left substantially unchanged.

Enteric fever has continued to prevail at Emsworth, from 5 to 21 cases annually having been notified since 1895. Between October 22nd and December 8th, 1902, nine Emsworth houses were invaded by fever, six of them before November 10th, and 13 persons were attacked. At about the same time oysters which had been stored in the ponds for different periods, some possibly for a few hours only, were supplied to the two mayoral banquets already referred to, which were held upon the same day. At Winchester there were 134 guests, of whom 62 (and a waiter) were attacked by illness of some kind, which became declared enteric fever in 11, of whom four died. At Southampton there were 132 guests, of whom 55 (and a waiter) were attacked by illness of some kind, which became declared enteric fever in 11, all of whom recovered. All the sufferers ate oysters, and there was no other article of food of which they all partook, or, indeed, against which any reasonable cause of suspicion could be substantiated.

The oysters supplied for both banquets were originally French, and the history of those sent to Southampton has been fully traced. On arrival from France they had been relaid in some unspecified English grounds, whence they were sent to Emsworth, and stored in the ponds there until wanted. They were of the kind known in the trade as "French, 1st quality;" but they were described on the *menu* of the Southampton dinner as "Huitres de Whitstable." It is at least generally believed that Whitstable oysters are effectually protected against sewage contamination, and it is therefore quite probable that the false description may have induced persons to partake of them who would not have done so if no description, or a true description, had been given. It does not appear whether the oyster dealer or the caterer was responsible for the falsehood; but it is significant that, in relation to an outbreak of enteric fever which occurred about the same time at Portsmouth, and which was attributed to oysters obtained from Emsworth, the dealer there at first denied, and was afterwards constrained to admit, that the oysters had been stored at Emsworth at all. He said at first that they had come direct from Whitstable without intermediate storage, but this assertion was ultimately shown to be untrue. In view of the magnitude of the oyster industry, and of the general importance of the question, it seems eminently desirable that the powers of the law should be tested, alike with reference to false representations by dealers, and to gross neglect on the part of local authorities; and that, if these powers were proved to be in either case insufficient, they should be amended without unnecessary delay.

